# MDCT-findings in patients with non-occlusive mesenteric ischemia (NOMI): influence of vasoconstrictor agents

**DOI:** 10.1007/s00330-023-09415-4

**Published:** 2023-01-24

**Authors:** Antoine Topolsky, Olivier Pantet, Lucas Liaudet, Christine Sempoux, Alban Denys, Jean-François Knebel, Sabine Schmidt

**Affiliations:** 1 Department of Diagnostic and Interventional Radiology, Lausanne University Hospital (CHUV) and University of Lausanne (UNIL), Rue du Bugnon 46, 1011 Lausanne, Switzerland; 2 Service of Adult Intensive Care Medicine and Burns, Lausanne University Hospital (CHUV) and University of Lausanne (UNIL), Lausanne, Switzerland; 3 Service of Clinical Pathology, Lausanne University Hospital (CHUV) and University of Lausanne (UNIL), Lausanne, Switzerland

**Keywords:** Intensive care unit, Mesenteric ischemia, Multidetector computed tomography, Vasoconstrictor agents

## Abstract

**Objectives:**

To evaluate the influence of vasoconstrictor agents (VCAs) on signs of vasoconstriction and bowel ischemia on MDCT detected in patients with non-occlusive mesenteric ischemia (NOMI).

**Methods:**

This 8-year single-center retrospective study consecutively included all patients with histopathologically proven NOMI who underwent MDCT ≤ 48 h prior to surgical bowel resection. Two blinded radiologists jointly reviewed each examination for signs of bowel ischemia, abdominal organ infarct, mesenteric vessel size and regularity, and ancillary vascular findings. VCA administration (length and dosage), clinical and biochemical data, risk factors, and outcomes were retrieved from patients’ medical records. Subgroup comparisons were performed.

**Results:**

Ninety patients were included (59 males, mean age 65 years); 40 (44.4%) had received VCAs before MDCT. Overall mortality was 32% (*n* = 29), with no significant difference between the two groups. In patients treated with VCAs, the calibre of the superior mesenteric artery (SMA) was smaller (*p* = 0.032), and vasoconstriction of its branches tended to be more important (*p* = 0.096) than in patients not treated with VCAs. The presence and extent of bowel ischemia did not significantly correlate with VCA administration, but abdominal organ infarcts tended to be more frequent (*p* = 0.005) and involved more organs (*p* = 0.088). The VCA group had lower mean arterial pressure (*p* = 0.006) and lower hemoglobin levels (*p* < 0.001). Several biomarkers of organ failure and inflammation, differed significantly with VCA use, proving worse clinical condition.

**Conclusions:**

MDCT demonstrates more severe SMA vasoconstriction and tends to show increased abdominal organ infarcts after VCA administration in NOMI patients compared to NOMI patients not treated with VCAs.

**Key Points:**

*• In critically ill patients with NOMI, MDCT demonstrates VCA support via increased vasoconstriction of the main SMA and its branches.*

*• VCA administration in NOMI patients tends to contribute to the development of organ infarcts, as shown on MDCT.*

*• An important degree of vasoconstriction in NOMI patients may indicate insufficient resuscitation and, thus, help clinicians in further patient management.*

## Introduction

Acute mesenteric ischemia (AMI) is a common and life-threatening complication in critically ill patients, especially intensive care units (ICUs). AMI has a high mortality rate of 52–60% [1, 2]. AMI is usually suspected in the presence of acutely deteriorating clinical conditions associated with digestive symptoms and laboratory findings, such as increasing serum lactate [3, 4].

Non-occlusive mesenteric ischemia (NOMI) is a subtype of AMI and must be distinguished from obstructive intestinal ischemia. NOMI accounts for 20–30% of all cases with acute bowel ischemia [5, 6] and is caused by splanchnic hypo-perfusion due to various conditions, such as cardiogenic shock, septic shock, dehydration, and hypotension. These states of hypo-perfusion activate a sympathetic response, resulting in increased cardiac output and further arterial mesenteric vasoconstriction. Classical risk factors include myocardial infarction; congestive, rhythmic, or valvular heart disease; major surgery, renal failure; and dialysis, all of which may typically be present in ICU patients [5–7].

The diagnostic criteria for NOMI include patency of both mesenteric arteries and veins, ischemic spots in several non-consecutive bowel segments over a wide area, and ischemic intestinal damage without fibrin plugs in the small veins [8, 9].

NOMI has the grimmest outcome of all subtypes of AMI, with an overall mortality rate of 70–90% [5, 10]. Therefore, immediate, and accurate identification of patients suffering from NOMI is important, and early radiological signs must not be missed.

Diagnosis of NOMI may be even more challenging than that of occlusive AMI. Clinical signs are non-specific, and abdominal pain is present in only 75% of NOMI patients [3, 7, 9]. Routine blood markers, such as leukocytosis and increased serum lactate are non-specific, but more specific and promising markers, such as intestinal fatty-acid binding protein (I-FABP), alpha-glutathione S-transferase (GST) and D-lactate, have not been consistently adopted in the clinical practice [5, 7].

Digital subtraction angiography (DSA) has been considered the method of choice to confirm the diagnosis of NOMI [7, 11]. Siegelman et al [12] defined the angiographic criteria for mesenteric arterial vasoconstriction as narrowing at the origins of multiple branches of the superior mesenteric artery (SMA), irregularities in the intestinal branches (i.e., “beading” sign or “string-of-sausage” sign), spasm of the arcades, and impaired filling of intramural vessels. However, in current daily practice, including our routine, patients suspected of NOMI are usually first investigated by MDCT, mainly due to the excellent spatial resolution inherent in modern MDCT machines [13, 14]. Moreover, unlike DSA, MDCT can simultaneously show any bowel wall or mesenteric sign directly or indirectly indicating AMI [4, 5, 15]. However, these signs may be subtle or present only at an advanced stage of NOMI; therefore, they are sometimes overlooked.

Vasoconstrictor agents (VCAs), such as (nor-)adrenaline, are commonly used to stabilize hemodynamically unstable patients, and the intensity of such therapy has been associated with an increased risk of NOMI [16]. We therefore addressed the hypothesis that the use and the dosage of these VCAs might influence the MDCT features we detect in these patients.

To the best of our knowledge, the relationship between the severity of vascular vasoconstriction and intestinal findings on MDCT in NOMI patients has not yet been described.

## Material and methods

### Patients

This was a single-center retrospective study prepared to conform to the Strengthening the Reporting of Observational Studies in Epidemiology (STROBE) guidelines [17].

**Fig. 1:**
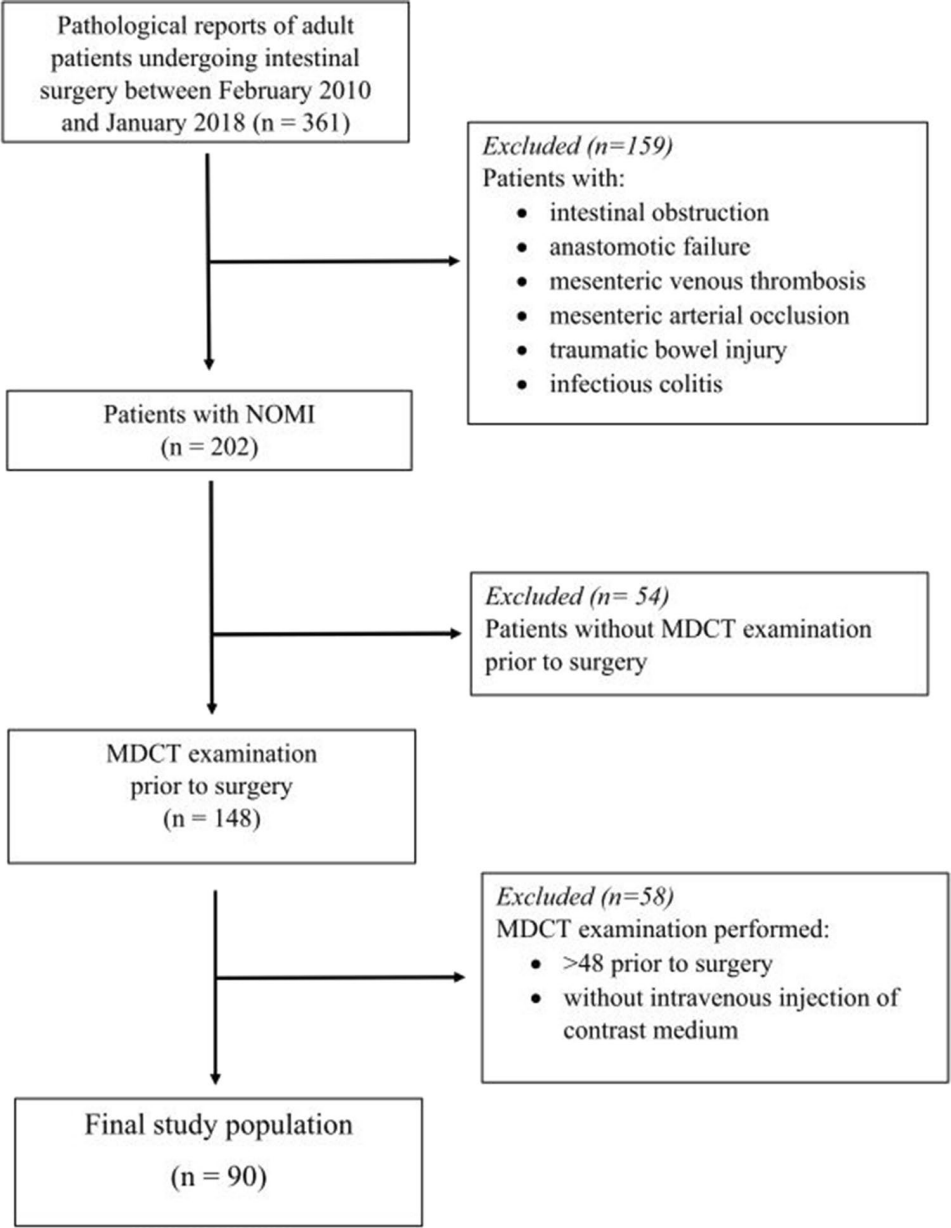
Flow chart showing patients’ inclusion

The study protocol (no 2018–00,464) was approved by our institutional ethics committee. Patients’ active consent was waived. Patient inclusion is shown in Fig. 1. Our final study population consisted of 90 consecutive patients who underwent MDCT examination ≤ 24 h prior to surgery. In each of our patients, bowel resection was performed, and the pathologist confirmed NOMI according to the histopathological definition described previously [8, 9]. Thus, histopathology was our reference standard for all patients.

### MDCT

MDCT examinations were performed on a 64-detector row CT machine (Lightspeed VCT; 64 Pro, GE Healthcare) from 2010 to 2015, and a 256-detector row CT machine (Revolution, GE Healthcare) from 2016 to 2018. The imaging protocol included the whole abdomen and pelvis (diaphragm to pubic symphysis, 120 kV, 300–400 mA, table speed 55 mm per rotation [0.8 s], pitch 1.375). The number of acquired abdominal scans (native, arterial, and/or portal phase) was variable. After a non-enhanced phase in 41 patients (2.5/2 mm reconstructed axial slices), we intravenously injected the iodinated contrast medium Accupaque® (Iohexol, 300 mgl/mL; GE Healthcare, volume in mL = body weight + 30 mL) at a flow rate of 4 mL/s for an arterial phase (25 s, 1.25/1 mm reconstructed axial slices) in 63 patients and a venous phase (80 s, 2.5/2 mm reconstructed axial slices) in 84 patients. We used the iterative reconstruction algorithm ASIR and automatic tube current modulation in all three axes (SmartmA).

### Image analysis

**Fig. 2:**
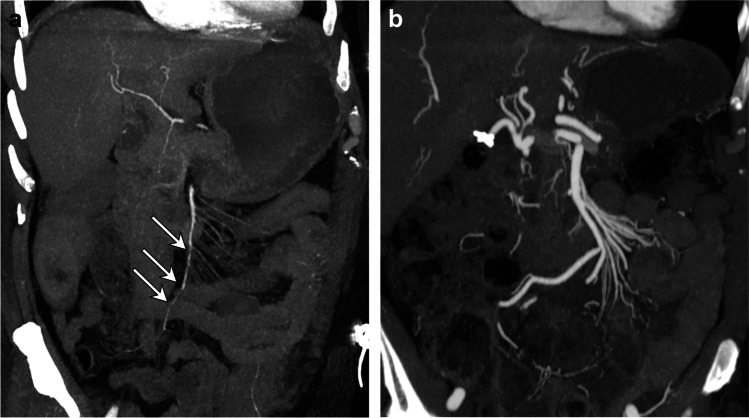
Coronal maximum intensity projection MDCT images (**a** and **b**) in two different NOMI patients show severe spasm of the main SMA and its branches (**a**, white arrows) in the patient receiving VCA support, unlike in the patient not receiving VCA support (**b**). **a** Coronal MDCT image of a 60-year-old man with NOMI in the ICU for post-surgical care with septic shock who received VCA support. Immediate laparotomy revealed ischemia of the terminal ileum and right colic angle. **b** Coronal MDCT image of an 82-year-old woman with NOMI presenting to the emergency department in shock with abdominal pain and who did not receive VCAs. Immediate laparotomy revealed ischemia of 20 cm of the small bowel

**Fig. 3:**
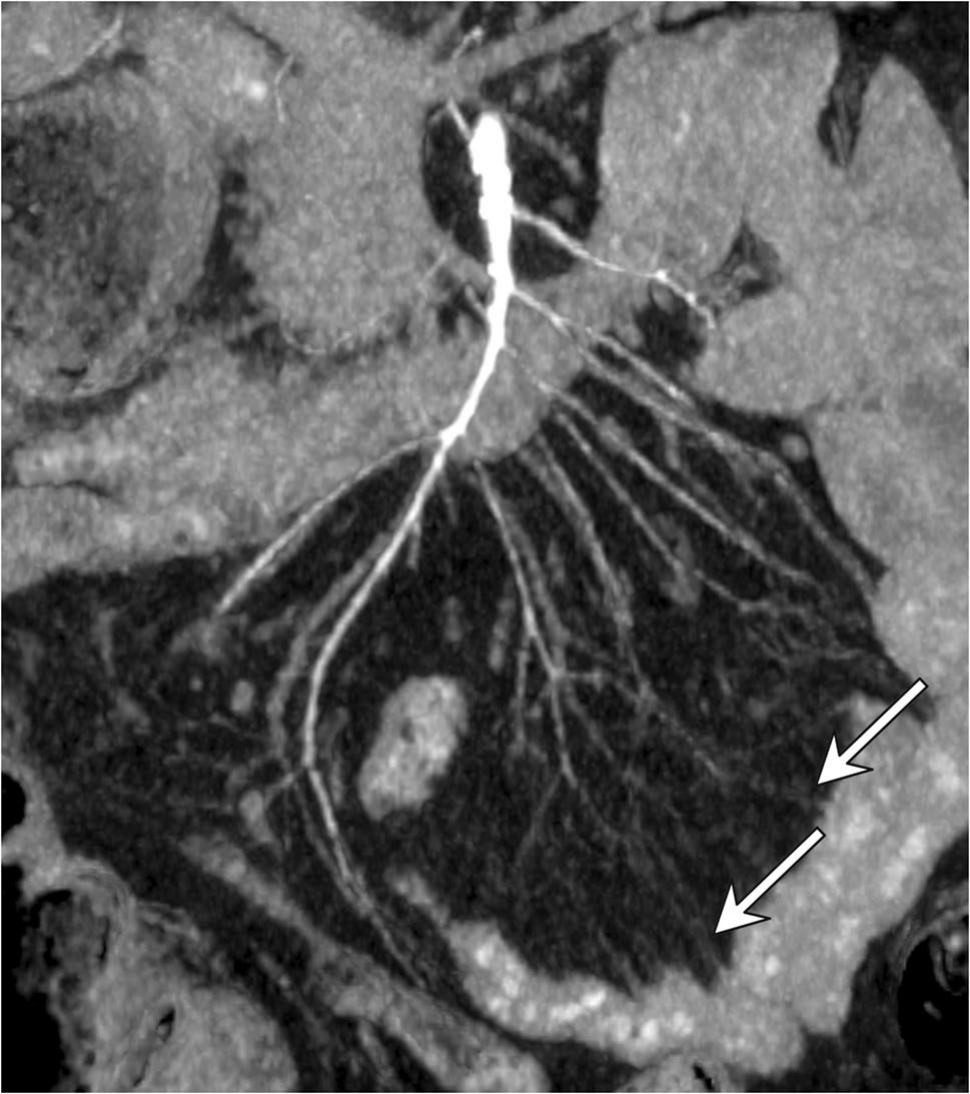
Coronal maximum intensity projection MDCT image shows severe vasoconstriction and irregularity of the main SMA and its branches, as well as spasm of the mesenteric arcades (white arrows) in an 80-year-old male patient in the ICU for post-surgical care with hemorrhagic shock receiving VCA support. Immediate laparotomy revealed necrosis of two ileal segments, each 40 cm in length

Two authors with 15 and 5 years of experience in abdominal imaging, respectively, who were blinded to the clinical data and the patients’ outcomes, jointly reviewed all MDCT images (Figs. 2 and 3) using a picture archiving and communication system workstation (Carestream Vue, version 11.4; Carestream Health). The examinations were displayed in soft tissue standard kernel. Windowing was modified as needed to optimize vessel visualization or detection of pneumatosis.

Our image analysis included vascular and extravascular signs (Table 1).

**Table 1:** Radiological findings, anamnestic data, and other parameters assessed in each patient

Radiological vascular findings	Vessels—mean diameter	Celiac trunk SMA (proximal, middle, distal) IMA SMV
	SMA spasm—extension	Main artery Main branches Arcades
	Vessels	Calcified atherosclerosis Aortic prosthesis Aortic aneurysm/dissection IABP LVAD
	Portal venous gas—distribution	Mesenteric arcade veins Segmental mesenteric veins Superior mesenteric veins Extrahepatic portal vein Intrahepatic portal veins
Radiological extravascular findings	Bowel	Wall hematoma Wall thickening Intestinal pneumatosis Decreased mural contrast enhancement Mural contrast hyperenhancement Luminal dilatation
	Peritoneal cavity	Mesenteric fat stranding Free fluid Extradigestive air
	Liver, spleen, kidney	Parenchymal infarct
Anamnestic data	Risk factors	Atrial fibrillation Heart disease Heart failure Pulmonary hypertension COPD Diabetes Obesity Cirrhosis Kidney failure
	Previous surgery (< 21 days)	Cardiovascular Thoracic (non-cardiovascular) Abdominal (non-vascular)
	Non-surgical previous event (< 21 days)	Cardiorespiratory arrest Burn Trauma
Hemodynamic parameters	MAP (mmHg) CVP (cmH_2_O)	
Laboratory parameters	Hb (g/L) WBC (G/L) Arterial pH pCO_2_ (mmHg) Serum lactate (mmol/L) CRP (mg/L) Procalcitonin (µg/L) ASAT (U/L) ALAT (U/L) LDH (U/L) Creatinine (µmol/L) BUN (mmol/L) CK (U/L) CK-MB (U/L)	
Medical treatment	Vasoconstrictor agents	Norepinephrine (mg) Epinephrine (mg) Vasopressin (mg)
	Other	Hydrocortisone (mg)

*SMA* superior mesenteric artery, *IMA* inferior mesenteric artery, *SMV* superior mesenteric vein, *IABP* intra-aortic balloon pump, *LVAD* left ventricular assist device, *COPD* chronic obstructive pulmonary disease, *MAP* mean arterial pressure, *CVP* central venous pressure, *Hb* hemoglobin, *WBC* white blood count, *CRP* C-reactive protein, *ASAT* aspartate aminotransferase, *ALAT* alanine aminotransferase, *LDH* lactate dehydrogenase, *BUN* blood urea nitrogen, *CK* creatinine kinase, *CK-MB* creatinine kinase myocardial band

Vascular findings were assessed using the arterial phase or portal venous phase. The vessels were measured as follows: celiac trunk, mean diameter 1 cm after the origin on axial and sagittal multi-planar reconstruction (MPR) images; SMA, mean diameter 1 cm after the origin, mean diameter after the origin of the inferior pancreaticoduodenal branch, and mean diameter after the origin of the ileocecal branch on axial curved MPR; and inferior mesenteric artery (IMA) patency and mean diameter 1 cm after the origin on axial and sagittal MPR. The spasm of the SMA was evaluated on coronal MIP (10.00 mm slabs), and its location along the vessel (main artery, main branches, and arcades) was documented. The presence of calcified atherosclerosis was evaluated using a visual qualitative scale (0 = absent, 1 = subtle calcifications, 2 = moderate calcifications, 3 = extensive calcifications). The presence of ancillary vascular findings, such as aortic prosthesis, left ventricular assist device (LVAD), or intra-aortic balloon pump (IABP), was also documented. Finally, the presence of portal venous gas, including the location was documented according to a pre-defined classification [18] as shown in Table 1**.**

Extravascular findings of mesenteric ischemia were assessed using all available phases [14, 15]. The bowel walls were assessed for hematoma, pathological thickening (> 3 mm), decreased enhancement, or abnormal hyperenhancement of the bowel mucosa (“target sign”) compared to nearby bowel loops. Intestinal pneumatosis, luminal dilatation, and associated mesenteric fat stranding were recorded. Intestinal dilatation was considered when the small bowel lumen measured > 3 cm and the colon lumen > 6 cm. The affected bowel loops were categorized into four locations: two groups of small bowel loops, situated either in the left or in the right hemiabdomen, the right colon (cecum, ascending colon, and transverse colon), and the left colon (rectum, sigmoid and descending colon up to the left colic flexure). The peritoneal cavity was assessed for the presence of any free fluid or extraintestinal air. Organ infarct was defined as parenchymal focal hypodensity of the liver, spleen, and/or kidneys and noted as either absent or present.

### Analysis of patients’ records

One author subsequently reviewed each electronic patient file using our two clinical workflow information systems, Soarian Clinical (Cerner Corporation) for in-patients and MetaVision (iMDsoft) for ICU patients.

Demographic information included age, gender, height, and body weight. Relevant comorbidities were defined as being either absent or present according to the clinical records and included cirrhosis (regardless of Child–Pugh staging), diabetes (regardless of type), obesity (body mass index [BMI] > 30 kg/m^2^), chronic obstructive pulmonary disease (COPD), regardless of stage), pulmonary hypertension, atrial fibrillation, any other heart disease, or heart failure (regardless of stage), and kidney failure (regardless of KDIGO staging). Laboratory and hemodynamic parameters, as well as medical treatment, were assessed as shown in Table 1**.** Relevant vital signs were recorded at three time points: at the time of the MDCT examination and 24 h and 48 h prior to MDCT. These signs included hemodynamic monitoring (mean arterial pressure [MAP], central venous pressure [CVP], arterial blood gas sampling hemoglobin [Hb], lactate dehydrogenase [LDH], pH, lactate, pCO_2_), inflammatory markers (white blood cell count [WBC]), C-reactive protein [CRP], procalcitonin), and markers of organ function markers (ASAT, ALAT, creatinine, BUN, CK, CK-MB).

Usage, type, and dosage of administrated VCAs were documented at the same three time points and averaged if necessary.

Finally, patient outcome was assessed.

Our reference standard was the patient’s histopathological report.

### Statistical analysis

Statistical analyses were performed using the software R (R Core Team) [19]. Data are presented as numbers and relative percentages. Continuous variables are presented as mean ± standard deviation (SD) and categorical variables as numbers or proportions. Between-group comparisons were performed using the unpaired student tests for continuous variables and the chi-squared test for categorical variables. The Pearson correlation coefficient was used to measure the linear relationship between two continuous variables. Statistical differences were considered significant for a *p* value < 0.05. For the problem of multiple testing, the *p* values of our radiological results were adjusted using the False Discovery Rate (FDR) methods [20].

## Results

### Patients

Of the final 90 patients, 34% were women (*n* = 31). The mean age was 65.4 ± 15.4 years (range, 16 – 92 years). The mean delay between MDCT and surgery was 0.6 days.

The overall mortality rate was 32% (*n* = 29), and the mean interval between MDCT and death was 14 days (range, 0 – 53 days). The main agent was norepinephrine (*n* = 40). As very few patients received epinephrine (*n* = 3), vasopressin (*n* = 3), or glucocorticoids (*n* = 6) in addition to norepinephrine, no supplementary analysis of the different VCAs or glucocorticoid administration was performed.

Forty (44%) patients received VCA support, and 50 (56%) did not.

The differences in demographics between the two groups are summarized in Table 2**.** The patients receiving VCA support had a higher BMI (*p* = 0.026) and a higher prevalence of cirrhosis (*p* = 0.003). We identified no other significant demographic difference between the two groups.

**Table 2:** Anamnestic data, risk factors, and outcomes in NOMI patients with and without VCA support

Variable	With VCA support (*n* = 40)	Without VCA support (*n* = 50)	*p* value
Age, years Women BMI, kg/m^2^	62.53 ± 15.2 13 27.7 ± 5.9	67.64 ± 15.2 18 24.8 ± 6.0	0.118 0.901 **0.026**
Aortic prosthesis	7	6	0.663
Aortic dissection/aneurysm	3	4	0.758
IABP	1	0	0.910
LVAD	1	0	0.910
Pulmonary hypertension	3	1	0.457
COPD	6	16	0.106
Diabetes	7	10	0.976
Cirrhosis	10	1	**0.003**
Atrial fibrillation	10	18	0.373
Heart disease	19	22	0.906
Heart failure	4	3	0.758
Kidney failure	6	9	0.924
Any surgery < 21 days	24	19	0.062
Death	16	13	0.236

Values are given as mean ± standard deviation or number of patients. Significant *p* values in boldface

*VCA* vasoconstrictor agent, *BMI* body mass index, *IABP* intra-aortic balloon pump, *LVAD* left ventricular assist device, *COPD* chronic obstructive pulmonary disease

### Imaging findings

The vascular and extravascular differences in MDCT findings between the two groups are summarized in Tables 3 and 4, respectively.

**Table 3:** Vascular imaging findings in NOMI patients with and without VCA support

Variable		With VCA support (*n* = 40)	Without VCA support (*n* = 50)	*p* value	Corrected *p* value (FDR)
Vascular mean diameter, mm^†^					
Proximal celiac trunk Proximal SMA Middle SMA Distal SMA Proximal IMA		5.5 ± 1.9 5.3 ± 1.2 4.9 ± 1.5 2.9 ± 1.1 2.9 ± 1.1	5.9 ± 1.8 5.9 ± 1.5 5.7 ± 1.2 3.2 ± 1.2 2.5 ± 1.1	0.3 **0.033** **0.004** 0.2 0.9	0.4 *0.09* **0.03** 0.3 0.9
SMA spasm	Any Main artery Main branches Arcades	27 4 19 26	23 3 15 21	**0.048** 0.7 0.1 **0.036**	*0.09* 0.8 0.2 *0.09*
Portal venous gas	Any Σ, segments	7 19	14 36	0.3 0.6	0.3 0.6

^†^ proximal = 1 cm after the origin; middle = after the origin of the inferior pancreaticoduodenal branch; distal = after the origin of the ileocolic branch

Values are given as mean ± standard deviation or the number of patients

Significant *p* values are in boldface, and *p* values showing a trend are written in italics

Σ = total number of affected segments or organs

*VCA* vasoconstrictor agent, *SMA* superior mesenteric artery, *IMA* inferior mesenteric artery, *FDR* false discovery rate

**Table 4:** Extravascular imaging findings in NOMI patients with and without VCA support

Variable		With VCA support (*n* = 40)	Without VCA support (*n* = 50)	*p* value	Corrected *p* value (FDR)
Extradigestive air		13	15	0.9	0.9
Free fluid		31	36	0.7	0.9
Mesenteric fat stranding		38	49	0.8	0.9
Organ infarct	Any Σ, organs	21 36	11 21	**0.005** **0.016**	*0.05* *0.09*
Intestinal pneumatosis	Any Σ, segments	13 21	14 17	0.8 0.6	0.9 0.8
Bowel wall hematoma	Any Σ, segments	4 5	4 5	0.7 0.4	0.9 0.6
Bowel wall thickening	Any Σ, segments	15 25	19 24	0.8 0.3	0.9 0.6
Decreased mural enhancement	Any Σ, segments	27 44	31 44	0.9 0.3	0.9 0.6
Mural hyperenhancement	Any Σ, segments	6 12	8 10	0.9 0.4	0.9 0.6
Luminal dilatation	Any Σ, segments	24 37	29 52	0.9 0.7	0.9 0.8

Σ = total number of affected segments or organs

Significant *p* values are in boldface, and *p* values showing a trend are written in italics

*FDR* false discovery rate

In the group of patients receiving VCAs, the SMA tended to have a significantly smaller diameter of 1 cm after the origin (mean 5.3 ± 1.1 mm, *p* = 0.09), and they had a significantly smaller diameter after the origin of the inferior pancreaticoduodenal branch (mean 4.9 ± 1.5 mm, *p* = 0.03) (Table 3). In the patient group without VCAs, the mean proximal diameter of the SMA measured 5.9 ± 1.5 mm, and the mean SMA diameter after the origin of the inferior pancreaticoduodenal branch measured 5.7 ± 1.2 mm.

Furthermore, SMA spasms tended to be more frequent (*p* = 0.09), and more often involving the arcades, occurring in 26 patients of the VCA group unlike in 21 patients without VCAs, 65%, *p* = 0.09).

There was no significant correlation between the VCA cumulative dose (mg/kg) over 24 h and over 48 h and the amount of vasoconstriction (*p* = 0.37 and *p* = 0.49, respectively) (Supplementary Table 1).

We found no significant difference in the atherosclerotic burden between the two groups (*p* = 0.725).

Of all analyzed extravascular findings (Table 4), only organ infarcts were significantly different between the two groups, with the patients receiving VCA tending to have both a higher prevalence of (*n* = 21, 52%, *p* = 0.05) and a higher number of organ infarcts (Σ = 36, *p* = 0.09), unlike the patients without VCAs (*n* = 11 and Σ = 36, respectively).

Neither the presence nor extent of acute ischemic bowel involvement differed significantly between the two groups.

### Clinical and laboratory findings

The clinical and laboratory findings in our two groups are shown in Table 5.

**Table 5:** Clinical and laboratory findings on the day of MDCT examination in NOMI patients with and without VCA support

Variable	Results (*n*)^†^	With VCA support	Without VCA support	*p* value
MAP, mmHg	77	73.9 ± 13.9	83.7 ± 16.9	**0.006**
CVP, cmH_2_O	27	12.0 ± 4.2	11.0 ± 4.2	0.7
Arterial pH	79	7.3 ± 0.1	7.3 ± 0.1	0.1
Base excess, mmol/L	79	–6.7 ± 6.6	–4.8 ± 6.4	0.2
Lactate, mmol/L	79	4.0 ± 4.0	3.0 ± 3.9	0.3
pCO_2_, mmHg	79	41.5 ± 11.8	38.5 ± 9.1	0.2
Hb, g/L	88	94.9 ± 16.7	110.8 ± 24.7	** < 0.001**
Leukocytes, G/L	88	19.6 ± 11.95	13.6 ± 7.8	**0.005**
CRP, mg/L	77	195.0 ± 101.2	109.3 ± 112.1	** < 0.001**
Procalcitonin, µg/L	24	19.4 ± 24.0	5.4 ± 8.1	0.1
ASAT, U/L	65	712.2 ± 1848.5	187.0 ± 725.2	0.1
ALAT, U/L	65	282.1 ± 779.1	56.4 ± 122.2	0.09
Creatinine, µmol/L	87	181.2 ± 120.1	121.2 ± 99.9	**0.012**
LDH, U/L	21	1507.7 ± 2246.7	451.7 ± 598.2	0.2
CK, U/L	49	1544.2 ± 2832.0	1016.6 ± 2452.3	0.5
BUN, mmol/L	57	18.87 ± 15.7	11.6 ± 6.4	**0.036**

Values are mean ± standard deviation unless otherwise noted. Significant *p* values are in boldface. † number of patients with results available for the said variable

*VCA* vasoconstrictor agent, *MAP* mean arterial pressure, *CVP* central venous pressure, *Hb* hemoglobin, *CRP* C-reactive protein, *ASAT* aspartate aminotransferase, *ALAT* alanine aminotransferase, *LDH* lactate dehydrogenase, *CK* creatine kinase, *BUN* blood urea nitrogen

On the day of the MDCT examination, MAP values were obtained for 77 patients (85%) and CVP values for 27 patients (30%). In patients receiving VCAs, MAP was significantly lower (mean 73.9 ± 13.9 mmHg, *p* = 0.006) whereas CVP showed no significant difference (mean 12.0 ± 4.2 cmH_2_O, *p* = 0.7).

As for the laboratory tests obtained on the day of the MDCT examination, there were no significant differences between the two groups in arterial pH, base excess, lactate, pCO2, procalcitonin, ASAT, ALAT, LDH, or CK. However, patients receiving VCAs had significantly lower Hb levels (mean 94.9 ± 16.7 g/L, *p* < 0.001), higher inflammatory markers such as leukocytes (mean 19.6 ± 11.95 g/L, *p* = 0.005), and CRP (mean 195.0 ± 101.2 mg/L, *p* < 0.001), and higher kidney failure markers such as creatinine (mean 181.2 ± 120.1 µmol/L, *p* = 0.012) and BUN (mean 18.87 ± 15.7 mmol/L, *p* < 0.036).

In patients receiving VCAs, leukocytes were significantly higher at 48 (mean 17.6 ± 8.7 g/L, *p* < 0.002) and 24 h (mean 20.9 ± 8.7 g/L, *p* < 0.003) prior to the MDCT examination (Table 5). The same was true for creatinine levels, which were higher both at 48 h (mean 149.5 ± 98.9 µmol/L, *p* < 0.042) and 24 h (mean 153.8 ± 95.7 µmol/L, *p* < 0.015) prior to MDCT. Such consistency was not observed for MAP, Hb, CRP, and BUN.

## Discussion

Our study including 90 patients with pathologically proven NOMI revealed the additional influence of VCA support on arterial vasoconstriction and the extent of abdominal organ infarcts. Nakamura et al [10] previously showed that the median SMA diameter is significantly smaller in NOMI patients (6.0 mm) than in controls (7.6 mm). We could further distinguish between NOMI patients with VCA support and NOMI patients without VCA support by demonstrating by MDCT an even smaller mean proximal and middle SMA diameter in the former (5.3 mm and 4.9 mm, respectively) than in the latter (5.9 mm and 5.7 mm, respectively). In addition, we could show that spasms of the SMA tend to be more frequent, especially in the arterial arcades, in patients treated with VCAs than those that were not.

The greater extent of abdominal organ infarcts we observed in the VCA-treated group seems to be the direct consequence of more severe splanchnic vasoconstriction caused by the VCA support. Systemic blood pressure is equal to cardiac output multiplied by systemic resistance, so clinicians may overestimate cardiac output and be falsely reassured by normal blood pressure values when they have massively increased resistance by adding VCAs without having properly corrected the cardiac output.

The presence and extent of bowel wall features indicating acute intestinal ischemia (i.e., decreased mural enhancement and intestinal pneumatosis), and the presence of portal venous gas did not differ significantly between the two groups. Indirect signs of acute intestinal ischemia, such as free fluid, mesenteric fat stranding, or even extradigestive air, were also not significantly different. Therefore, these signs may not be directly influenced by the medically induced vasoconstriction itself but are mainly determined by other pathophysiological factors that are beyond our analysis.

A comparison of the epidemiological risk factors only yielded two significant differences: patients treated with VCAs had a higher prevalence of cirrhosis and a higher BMI. Cirrhotic patients are known to have immune dysfunction, altered inflammatory responses, and circulatory dysfunction, all of which contribute to a greater susceptibility to septic shock [21]. As previously shown by Durst et al [22], cirrhotic patients in septic shock more frequently require VCA support than non-cirrhotic patients, and for an increased duration. Thus, the higher BMI observed in patients receiving VCAs may merely reflect the higher clinical and hemodynamic vulnerability of these patients, though recent studies have shown a paradoxical survival benefit of obesity in critically ill patients in the ICU [23, 24]. Nevertheless, our study did not show any significant difference in mortality between our two groups. Furthermore, the overall mortality rate in our NOMI patients was 32%, which is lower than previously reported [5, 10].

Patients receiving VCA support had elevated biomarkers of kidney failure (creatinine, BUN). Furthermore, they had significantly lower MAP and Hb levels. These findings point to the hypothesis of insufficient resuscitation, with persistently reduced systemic blood flow and oxygen transport, but this appears not supported by the lack of any differences in arterial blood lactate concentration, a surrogate marker of impaired tissue oxygenation. Impaired microcirculatory blood flow in response to VCA administration may represent an additional hypothesis, owing to the known influence of norepinephrine on the microcirculation [25], but this issue remains speculative, given the absence of detailed hemodynamic studies in our patients.

Patients receiving VCA support had significantly higher inflammatory biomarkers (leukocytes and CRP). A high leukocyte count has been reported to be an early indicator of NOMI, and a reduced Hb level is a risk factor for acute intestinal ischemia leading to reduced transport of oxygen to the gut [16, 26]. However, with these arguments, the differences between our two groups, including a possible role of VCAs, are still not explained. Thus, we simply attribute them to the worse clinical condition of NOMI patients receiving VCAs, with later recognition of mesenteric ischemia and the more frequent onset of septic shock requiring VCAs.

Our study has several limitations. First, our gold standard of histopathologically proven NOMI is a selection bias, as it may exclude a patient with MDCT findings of NOMI in whom abdominal surgery was withheld, which can lead to an underrepresentation of milder cases or more critical illness. Second, our study was retrospective and subject to documentation bias, such as infrequent or missing laboratory markers, which may explain the variation in the significance of relevant clinical and laboratory markers over time. Furthermore, for simplicity, we have excluded very rarely used VCAs, such as epinephrine (*n* = 3), vasopressin (*n* = 3), and glucocorticoids (*n* = 6). Similarly, the low prevalence of some specific MDCT signs may have prevented us from detecting significant differences between our two groups. Finally, we did not extract cardiac output values that could support the hypothesis that NOMI patients with vasoconstrictors are more often in states of low cardiac output.

In conclusion, our study showed the usefulness of MDCT for detecting the additional influence of VCA support on arterial vasoconstriction and the extent of abdominal organ infarcts in NOMI patients. The radiologist needs to be aware of these vascular consequences, which may help in reading MDCT in critically ill patients with suspected acute intestinal ischemia, especially when direct or indirect bowel signs of acute ischemia are not (yet) visible. In addition, an important degree of vasoconstriction observed in these patients may indicate insufficient resuscitation and, thus, help clinicians in further patient management.

## Supplementary Information

Below is the link to the electronic supplementary material.

Supplementary file1 (DOCX 79 KB)

## Abbreviations

ALAT: Alanine aminotransferase
AMI: Acute mesenteric ischemia
ASAT: Aspartate aminotransferase
BMI: Body mass index
BUN: Blood urea nitrogen
CK: Creatine kinase
COPD: Chronic obstructive pulmonary disease
CRP: C-reactive protein
CVP: Central venous pressure
DSA: Digital subtraction angiography
IABP: Intra-aortic balloon pump
ICU: Intensive care unit
IMA: Inferior mesenteric artery
LDH: Lactate dehydrogenase
LVAD: Left ventricular assist device
MAP: Mean arterial pressure
MDCT: Multi-detector computerized tomography
MIP: Maximum intensity projection
MPR: Multi-planar reconstruction
NOMI: Non-occlusive mesenteric ischemia
SMA: Superior mesenteric artery
VCA: Vasoconstrictor agents
WBC: White blood cell

## Declarations

### Guarantor

The scientific guarantor of this publication is Sabine Schmidt Kobbe.

### Statistics and biometry

One of the authors, i.e., Jean-Francois Knebel, has significant statistical expertise.

### Methodology

• retrospective

• cross-sectional study

• observational

• performed at one institution
