# Supplementary material for: MDCT-findings in patients with non-occlusive mesenteric ischemia (NOMI): influence of vasoconstrictor agents
Source: Eur Radiol. 2023 Jan 24;33(5):3627–37. doi: 10.1007/s00330-023-09415-4 (PMC10121529; doi:10.1007/s00330-023-09415-4)
Supplement: Supplementary file 1 — Supplementary file1 (DOCX 79 KB) [file 330_2023_9415_MOESM1_ESM.docx]

**Supplementary Table 1**. Bivariate correlation results between VCA and vascular findings

| **Variable 1** | **Variable 2** | **Coefficient, r** | **P-value** |
| --- | --- | --- | --- |
| Norepinephrine (Σ over 48h), mg | Vascular mean diameter, mm^†^ |  |  |
|  | Proximal celiac trunk  Proximal SMA  Proximal IMA | -0.150  -0.045  -0.172 | 0.357  0.781  0.390 |
| Norepinephrine (Σ over 48h), mg/kg | Vascular mean diameter, mm |  |  |
|  | Proximal celiac trunk  Proximal SMA  Proximal IMA | -0.125  -0.111  -0.258 | 0.443  0.495  0.194 |
| Norepinephrine (Σ over 12h), mg | Vascular mean diameter, mm |  |  |
|  | Proximal celiac trunk  Proximal SMA  Proximal IMA | -0.174  -0.061  -0.105 | 0.283  0.710  0.602 |
| Norepinephrine (Σ over 12h), mg/kg | Vascular mean diameter, mm |  |  |
|  | Proximal celiac trunk  Proximal SMA  Proximal IMA | -0.152  -0.145  -0.224 | 0.349  0.371  0.261 |

Σ = cumulative dosage before MDCT examination

† proximal = 1 cm after the origin

VCA – vasoconstrictor agent, SMA – superior mesenteric artery, IMA – inferior mesenteric artery
